# Factors Associated with the Uptake of Genetic Testing for Cancer Risks: A Pathway Analysis Using the Health Information National Trends Survey Data

**DOI:** 10.3390/life12122024

**Published:** 2022-12-04

**Authors:** Xiangning Dong, Jingxian Huang, Yanze Yi, Lanwei Zhang, Tenglong Li, Ying Chen

**Affiliations:** 1 Department of Biological Sciences, School of Science, Xi’an Jiaotong-Liverpool University, Suzhou 215000, China; 2 Wisdom Lake Academy of Pharmacy, Xi’an Jiaotong-Liverpool University, Suzhou 215000, China

**Keywords:** pathway analysis, cancer risk, health behavior, genetic testing uptake, information source, perception of cancer, attitude towards cancer, psychosocial factor, HINTS, cross-sectional study

## Abstract

Our study aimed to identify pathways from the source of information to the uptake of cancer genetic testing, with consideration of intermediate variables including perceptional, attitudinal and psychosocial factors. We used the Health Information National Trends Survey (2020 database) and constructed a structural equation model for pathway analysis (using SPSS version 24). Variables for socio-demographic, lifestyle and health information were also collected and used for confounding adjustment. A total of 2941 participants were analyzed (68.5%, non-Hispanic white; 59.7%, females; 58 years, median age; and 142 (4.8%) had undertaken genetic testing for cancer risk previously). Our pathway analysis found that only information from particular sources (i.e., healthcare providers and genetic counsellors) had positive and significant effects on people’s perceptions of cancer regarding its prevention, detection and treatment (standardized β range, 0.15–0.31, all *p*-values < 0.01). Following the paths, these perceptional variables (cancer prevention, detection and treatment) showed considerable positive impacts on the uptake of genetic testing (standardized β (95% CIs): 0.25 (0.20, 0.30), 0.28 (0.23, 0.33) and 0.12 (0.06, 0.17), respectively). Pathways involving attitudinal and psychosocial factors showed much smaller or insignificant effects on the uptake of genetic testing. Our study brings several novel perspectives to the behavior model and may underpin certain issues regarding cancer risk genetic testing.

## 1. Introduction

According to the International Agency for Research on Cancer, cancer ranks as the second largest cause of premature mortality worldwide, and in 2020 alone it cost approximately 10 million lives [1]. It imposes tremendous physical and mental burdens on patients and their families [2,3,4,5,6]. Early diagnosis and treatment have become the main strategy for cancer management at both the individual and population level, as it is shown to lead to potential better outcomes or a possible cure [7,8,9,10]. With the progress of genomic research, especially the Human Genome Project [11,12], genetic testing has become a popular screening method for hereditary cancer syndromes, which can lead to recommendations for earlier screening or prophylactic surgery [13,14,15,16,17,18,19]. It is particularly helpful for those with a personal and family history of cancer. Genetic testing, along with other information, facilitates decision making in cancer risk evaluation and prediction [20,21,22,23]. Those who have low risks of developing cancer could also benefit from a proper education built upon evidence acquired from their genetic testing [24,25].

When introducing genetic testing into healthcare practice, the understanding of the relevant knowledge by the public, as well as psychosocial and attitudinal responses, are the driving forces for its adoption and uptake [26,27,28]. Using structural equation modelling, Wade and colleagues provided evidence that certain health-related behaviors, such as worry and perception, may function as mediators in the association pathways between socio-demographic characteristics (e.g., age, level of education and race/ethnicity) and testing intention [29]. Some common health-related behaviors, such as cognitive attitude, uncertainty avoidance and perception of risk, studied in the ‘Planned Behavior Theory’, ‘Protection Motivation Theory’ and ‘Health Trust Model’, were reported to be significant predictors of testing intention [30,31,32]. Meanwhile, psychosocial factors, such as avoidance of cancer risk information, were found to be negatively associated with cancer prevention behavior (e.g., screening for colon cancer) [33]. Psychological characteristics were also reported to have an important impact on the decision making process to undergo genetic testing [34,35,36,37].

Previous research suggested information exposure to media coverage could shape the public’s perception on genetic testing [38]. Advertising of particular genetic testing services was associated with elevated awareness and interest in genetic testing [39,40]. However, it may not necessarily result in an actual increase in the uptake rate [41,42]. Information from certain sources, for example the webpages of a genetic testing company, was found to have insufficient content for their consumers to make reasonable decisions [43]. Nevertheless, according to Keller et al., an expressed intention and attitude towards genetic testing does not reliably predict actual uptake activity [44].

Previous studies investigated several individual associations or pathways regarding the relationship between intention and uptake of genetic testing with their predictor factors, such as attitude and perception, psychological characteristics, and information sources [29,30,31,32,33,34,35,36,37,41,42,45,46]. However, to the best of our knowledge, there is still no research that integrates all the aforementioned factors in one study.

We hypothesize that information from particular media coverage would affect people’s perception of genetic testing. Together with other attitudinal and psychosocial factors, this would influence the uptake of genetic testing for high-risk cancer assessment. In this paper, we aim to use structural equation modelling (SEM) to explore the pathways from media sources to perceptional variables, and subsequently from perceptional, attitudinal and psychosocial variables to the uptake of high-risk cancer genetic testing. We believe the findings from our research would address certain issues regarding the uptake of genetic testing for cancer risks.

## 2. Materials and Methods

### 2.1. Study Population

We used data from the Health Information National Trends Survey (HINTS 5, Cycle 4), which was conducted between February and June 2020. The HINTS project is hosted by the National Cancer Institute (NCI), and has actively been collecting surveys from nationally representative samples in the USA since 2003 (https://hints.cancer.gov/, accessed on 18 November 2022). The surveys target non-institutionalized adults aged 18 and above. They are intended as a comprehensive assessment of the access and use of health information, as well as the public’s attitudes about cancer in terms of the following aspects: risk perception, vital prevention, early detection, diagnosis, and treatment. 

**Figure 1:**
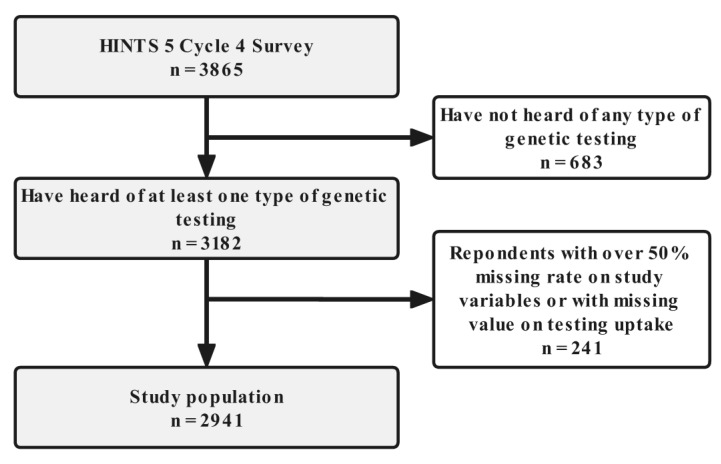
Flow diagram of the population included in this study.

The 4th cycle of HINTS 5 employed an equal-probability sampling method for household selection, and the Next Birthday Method for respondent selection from each household. The questionnaires were written in English or Spanish and distributed by mail. The survey in total targeted 10,531 individuals, with a response rate of 36.7% resulting in 3865 respondents who returned their answered questionnaires. The study sample used in our analysis (n = 2941), was a result of the selection procedure illustrated in Figure 1. Survey participants were asked whether they had heard of any types of genetic testing in the questionnaire, with those who responded ‘I have not heard of any of these types of genetic testing’ excluded first (n = 683). Respondents with over a 50% missing rate on study variables (as defined in the following section) or with missing values on the uptake of genetic testing were excluded second (n = 241).

### 2.2. Study Variable

**Figure 2:**
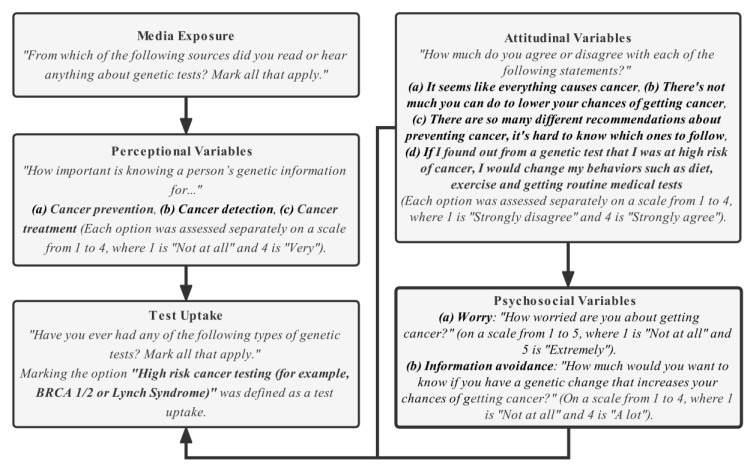
The conceptual framework and study variables, with arrows indicating the direction of possible effects.

Variables (along with the corresponding survey questions) used in this study for the conceptual planning and pathway construction are described in Figure 2. Important factors, such as media exposure (an information source about cancer genetic testing), perception, attitude, psychosocial factors, and the uptake of genetic testing were included. Additionally, variables on socio-demographics (including age, gender, BMI, race/ethnicity, marital status, education level, employment status, annual income, health insurance coverage, residential location (urban vs. rural), lifestyle (including cigarette smoking, alcohol drinking, and exercise activity) and health information (including general health score and personal and family cancer history) were also collected and used for mitigating potential confounding effects.

### 2.3. Statistical Analysis

The average proportion of missing data among studied variables was 4.7%. We employed the R package ‘rpart’ (Recursive Partitioning and Regression Trees, version 4.1.15, by Bell Laboratories, New Jersey, NJ, USA), a decision-tree based method, to impute those missing values on media exposure, perceptional variables, attitudinal variables, psychosocial factors, socio-demographics (including age, gender, BMI, race/ethnicity, marital status, education level, employment status, annual income, and health insurance coverage), and lifestyle (including cigarette smoking, alcohol drinking, and exercise activity).

We used Fisher’s Exact test to initially select the variables for media exposure and perception, which were significantly different between those who had undergone genetic testing and who had not. We also screened the socio-demographic variables and health indicators using a similar method and selected statistically significant variables for confounding adjustment. We then constructed SEM for pathway analysis. SEM is a multivariate statistical framework used to model complex relationships between directly and indirectly observed (latent) variables. The model-fitting was performed by IBM SPSS (version 24), which works with a combination of multiple single SEMs to implement pathway analysis. We used single headed arrows to build pathways from influential to affected variables. The conceptual pathways between the studied variables are exhibited in Figure 2. Generalized least squares estimates were used to estimate each parameter, including the covariances between variables, the standard errors of the covariances, the critical ratios, and the two-tailed *p*-values. The 95% confidence intervals (95% CIs) for all parameters were calculated using the bootstrapping method. Standardized regression coefficients (β) were reported, obtained by using data after standardization. Data standardization was calculated by first subtracting the mean of a variable from its raw value and then dividing it by its standard deviation.

A *p*-value of 0.05, two-tailed, was set as the threshold for statistical significance. Statistical analyses were conducted in R if not mentioned elsewhere.

## 3. Results

More than half of the study participants were non-Hispanic white (68.5%) and female (59.7%). The median age was 58 years (interquartile range 42–68 years). A summary of collected variables in socio-demographics, lifestyle factors, and health information is provided in Table 1. Approximately 5% (142/2941) of the study participants reported that they had undergone cancer genetic testing previously. Participants who were female, covered with health insurance, employed, and with a reported personal and family history of cancer, were found to be more likely to undertake genetic testing for cancer risks (Table 1).

Mass media were the most common sources of information about genetic testing reported by our study participants. Television (62.6%) and internet (49.1%) were listed as the top two sources of information, followed by papers (newspapers 15.2% and magazines 17.8%) and radio (16.4%) (Table 2). Social communications were also important sources: 32.7% of the participants had received information from social media; 37.0% from family members; and 28.4% from friends (Table 2). Information from health professionals was the least commonly reported source: 19.4% of the participants had obtained it from healthcare providers and 3.6% from genetic counsellors (Table 2). However, analyses showed that only exposure to information from healthcare providers (those who had genetic testing vs. those who had not, 59.9% vs. 17.3%, *p* < 0.01) and genetic counsellors (27.5% vs. 2.4%, *p* < 0.01) was positively associated with the uptake of genetic testing (Table 2). As the most common source of information, television was found to be negatively associated with the uptake of genetic testing (those who had genetic testing vs. those who had not, 51.4% vs. 63.2%, *p* < 0.01, Table 2).

With regards to the perceived importance of genetic information, the majority of participants approved of the importance for cancer prevention (‘a lot’ and ‘somewhat important’ categories combined, 80.4%), detection (85.7%), and treatment (79.5%). Participants who had a higher perceived importance of genetic information for cancer prevention, detection, and treatment, were more likely to report an uptake of genetic testing than those who had a lower perceived importance (Table 3).

**Figure 3:**
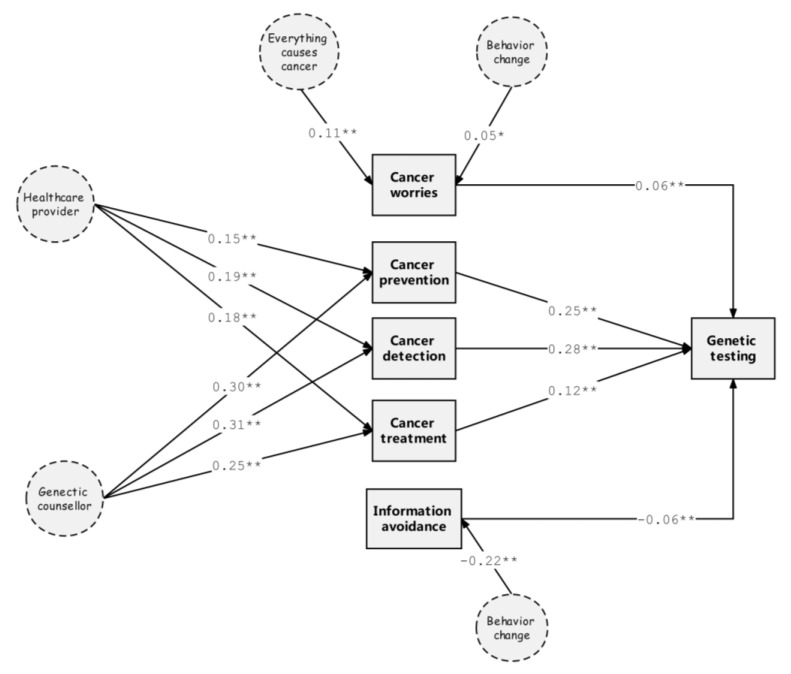
Pathway model for the uptake of cancer genetic testing (arrows indicate the conceptual route from influential to affected factors in particular pathways; values indicate the magnitude and direction of the effect of particular pathways; stars demonstrate the level of statistical significance: * *p* < 0.05 and ** *p* < 0.01). Results are adjusted for gender, occupation, insurance, family cancer history, personal cancer history, age group, race, education, and marital status).

Figure 3 summarizes the pathway model of influential variables for the uptake of genetic testing, where only statistically significant variables are included. Among the paths that led to the uptake of genetic testing, perception variables (cancer prevention, detection, and treatment) showed a considerable positive impact (standardized β (95% CIs): 0.25 (0.20, 0.30), 0.28 (0.23, 0.33) and 0.12 (0.06, 0.17), respectively), whereas the influence from psychosocial variables (cancer worries and information avoidance) were much smaller with either a positive or negative effect (standardized β (95% CIs): 0.06 (0.02, 0.09) and −0.06 (−0.09, −0.03), respectively). Perception variables were all strongly affected by information provided by health professionals (i.e., healthcare providers and genetic counsellors, Figure 3). Among the four attitudinal variables, only ‘behavior change’ had a significant negative effect on information avoidance (standardized β (95% CIs): −0.22 (−0.26, −0.17)), while also exhibiting a weak positive effect on cancer worries (standardized β (95% CIs): 0.05 (0, 0.09)). Additionally, an attitude towards ‘everything causes cancer’ had a significantly positive relationship with cancer worries (standardized β (95% CIs): 0.11 (0.07, 0.16)). Television was not significantly involved in the pathway model.

## 4. Discussion

This study has reported the public’s perspectives on the genetic testing for cancer risks. One particular novelty is that we have integrated information sources into the behavioral model for predicting the uptake of cancer risk genetic testing. This study advocates for a better understanding of the factors associated with the uptake of cancer risk genetic testing using a pathway analysis, which may underpin certain issues on the topic and may encourage a better atmosphere for public participation in this healthcare practice.

Information about cancer risk genetic testing from particular media types, as the original source of knowledge, was studied in our pathway analysis. This ‘media-perception-uptake’ pathway model should be considered as a meaningful contribution to the existing behavior models on genetic testing engagement, since our results show that only information expressed by certain sources have a positive impact. Healthcare providers and genetic counsellors were the only two sources of information that positively influenced individuals’ perceptions on cancer genetic testing in our analysis. However, the proportion of study participants receiving information about genetic testing from these two sources were small. In particular, only about 4% of participants received information from genetic counsellors (Table 2). It is thus important to proactively engage genetic counsellors in the education of cancer risk genetic testing [47]. However, gaps between the services that genetic counsellors can offer and the expectations for genetic testing products have been identified [48].

Direct to consumer genetic testing (DCGT) has become an emerging service that allows individuals to have their genetic background examined without having to consult an expert [49]. However, it was found that many DCGT offers do not meet a minimum set of quality criteria that is necessary for ensuring that adequate information and protection is given to customers against misleading interpretations of the need for, as well as the possible consequences of, genetic testing; most DCGT offers fail to provide proper information on the scientific evidence behind genetic testing services offered to customers (clinical validity and utility), and many of the companies offering genetic testing services via the internet do not include genetic counseling at all in their services [43]. In a follow-up study, approximately 15% of DCGT customers still contacted genetic counsellors, as they believed that such consultation would enhance their knowledge about the genetics and, in particular, the interpretations of their own testing results [50]. However, many surveyed genetic counsellors were reluctant to provide the genetic counseling service to the DCGT customers since they believed that such a service should be provided by the DCGT companies [43]. With the burden on genetic services, including the current topic of cancer risk genetic testing, there is an argument for the increased and adaptive use of genetic counsellors [51].

Our results also showed that television was the largest source of information for genetic testing (63%, Table 2). However, the effect of television on the perceived importance of genetic information was not statistically significant in our pathway model. According to Allen et al., exposure to television cancer advertisements rarely alleviates individual concerns about the risk of cancer or cancer prevention, as most advertisements focus on fear-based emotional appeal and cancer treatment rather than prevention [52]. In addition, we also observed little effect of internet, newspaper, magazine, radio, and social communications (social media, families and friends), as the source of information about genetic testing, on the uptake of cancer risk genetic testing. Our results suggest that the mass media itself, or the current presentation of knowledge on these platforms, may not be an effective method to sufficiently deliver information which could actually increase the uptake of genetic testing.

In the framework of pathway analysis, our results suggest that the studied attitudinal variables (i.e., ‘everything causes cancer’, ‘prevention is not possible’, ‘too many recommendations to follow’, and ‘behavior change’) are not directly associated with the uptake of cancer risk genetic testing. Instead, some variables (including ‘everything causes cancer’ and ‘behavior change’) are found to be associated with psychosocial variables, although some psychosocial variables are also found to be associated with the uptake of testing. This observation is generally consistent with the findings of a previous study which also used the HINTS database (of 2017) [53]. In a younger population group (aged 25–40 years old), however, attitudes towards undergoing genetic testing are identified as significant predictors of testing uptake [29]. Nevertheless, the attitudinal variables in our analysis are measurements more about general beliefs and attitudes towards cancer than exact attitudes towards genetic testing.

Results in our analysis suggest that some psychosocial variables (i.e., ‘cancer worries’ and ‘information avoidance’) are significantly involved in our pathway model influencing the uptake of genetic testing. However, a previous study, which also used the HINTS database but in a different cycle (2017), concluded that psychosocial variables were not associated with uptake [53]. In our study, a general concept of testing was used as it referred to all possible types of cancer-related genetic testing, whereas the previous study used a more specific concept where only testing for BRCA 1/2 and Lynch syndrome risks was included [53]. The current study, using an existing survey, demonstrates that ‘cancer worries’ and ‘information avoidance’ are two independent factors influencing the uptake of genetic testing through different pathways (Figure 3). However, the actual situation may be far more complicated. For example, ‘cancer worries’ could be the basis for certain types of ‘information avoidance’, such as doctor avoidance [54]. These two factors could be intertwined and have an interactive impact on the uptake of genetic testing within the same group of individuals.

This study has several limitations. It is a survey-based study, therefore information used for analysis is self-reported and the data is cross-sectional in nature. The HINTS 2020 is adapted to perform this pathway analysis, so the analysis is limited to the scale and concept of the variables collected in the survey. HINTS aims to collect nationally representative samples within the USA. However, its response rate is relatively low, therefore selection bias may exist. We constructed a behavior model to demonstrate the ‘media-perception-uptake’ pathway for the general population, but not all genetic testing services should be recommended for such people. Analysis for those recognized as potentially high-risk individuals from initial screening (e.g., based on age, BMI, smoking, alcohol use, exposed to certain agents, having certain symptoms, etc.) is required.

In conclusion, using the HINTS 2020 database, our study demonstrated the effective pathways regarding the factors associated with the uptake of cancer genetic testing, while estimating the size of these effects (direct or indirect) from certain media exposures, perceptional variables, attitudinal variables, and psychosocial variables on the testing uptake. We suggest that information about genetic testing from only professional sources (such as healthcare providers and genetic counsellors) would positively influence individuals’ perceptions on cancer genetic testing. Our pathway model also suggests that the perceived importance of genetic information on preventing, detecting, and treating cancer is the direct and strongest predictor for the uptake of genetic testing. Our study brings several novel perspectives to the behavior model and may underpin certain issues regarding the genetic testing for cancer risks. To confirm the results, studies with larger scales or more representative populations must be conducted, preferably in other countries besides the USA.

## Figures and Tables

**Table 1:** Population characteristics of all study participants and participants stratified by uptake of genetic testing.

Characteristics	Did Genetic Testing (n = 142)	Did Not (n = 2799)	Total (n = 2941)	*p* Value
Gender, n (%)				
Male	30 (21.1)	1154 (41.2)	1184 (40.3)	<0.01
Female	112 (78.9)	1645 (58.8)	1757 (59.7)	
Age group, n (%)				
18–34	10 (7.0)	408 (14.6)	418 (14.2)	
35–49	32 (22.5)	548 (19.6)	580 (19.7)	0.085
50–64	56 (39.4)	912 (32.6)	968 (32.9)	
65–74	30 (21.1)	619 (22.1)	649 (22.1)	
75 and above	14 (9.9)	312 (11.1)	326 (11.1)	
BMI category, n (%)				
Underweight	3 (2.1)	37 (1.3)	40 (1.4)	
Normal	51 (35.9)	852 (30.4)	903 (30.7)	0.340
Overweight	44 (31.0)	914 (32.7)	958 (32.6)	
Obesity	44 (31.0)	996 (35.6)	1040 (35.4)	
Marital status, n (%)				
Married/Living together	74 (52.1)	1545 (55.2)	1619 (55.0)	0.071
Divorced/Separated	50 (35.2)	761 (27.2)	811 (27.6)	
Single/Never married	18 (12.7)	493 (17.6)	511 (17.4)	
Location, n (%)				
Metropolitan	127 (89.4)	2459 (87.9)	2586 (87.9)	
Micropolitan	10 (7.0)	201 (7.2)	211 (7.2)	0.961
Small town	4 (2.8)	93 (3.3)	97 (3.3)	
Rural	1 (0.7)	46 (1.6)	47 (1.6)	
Race, n (%)				
Non-Hispanic White	100 (70.4)	1914 (68.4)	2014 (68.5)	
Non-Hispanic Black	20 (14.1)	397 (14.2)	417 (14.2)	0.920
Hispanic	17 (12.0)	360 (12.9)	377 (12.8)	
Non-Hispanic other	5 (3.5)	128 (4.6)	133 (4.5)	
Education, n (%)				
Less than High School	12 (8.5)	122 (4.4)	134 (4.6)	
High School	20 (14.1)	442 (15.8)	462 (15.7)	0.053
Some College	45 (31.7)	849 (30.2)	891 (30.3)	
Bachelor	30 (21.1)	813 (29.0)	843 (28.7)	
Post-Baccalaureate	35 (24.6)	576 (20.6)	611 (20.8)	
Occupation, n (%)				
Employed	76 (53.5)	1516 (54.2)	1592 (54.1)	
Unemployed	4 (2.8)	159 (5.7)	163 (5.5)	0.020
Retired	38 (26.8)	855 (30.5)	893 (30.4)	
Other	24 (16.9)	269 (9.6)	293 (10.0)	
Income, n (%)				
Less than USD 20,000	24 (16.9)	443 (15.8)	467 (15.9)	
USD 20,000 to <USD 35,000	11 (7.7)	301 (10.8)	312 (10.6)	0.843
USD 35,000 to <USD 50,000	16 (11.3)	328 (11.7)	344 (11.7)	
USD 50,000 to <USD 75,000	24 (16.9)	464 (16.6)	488 (16.6)	
USD 75,000 or More	67 (47.2)	1263 (45.1)	1330 (45.2)	
Insurance, n (%)				
Yes	141 (99.3)	2665 (95.2)	2806 (95.4)	0.021
No	1 (0.7)	134 (4.8)	135 (4.6)	
Smoke, n (%)				
Current	15 (10.6)	310 (11.1)	325 (11.1)	0.925
Former	37 (26.1)	690 (24.7)	727 (24.7)	
Never	90 (63.4)	1799 (64.3)	1889 (64.2)	
Moderate drink, n (%)				
Yes	106 (74.6)	2215 (79.1)	2321 (78.9)	0.241
No	36 (25.4)	584 (20.9)	620 (21.1)	
Sufficient exercise per week, n (%)				
Yes	55 (38.7)	1074 (38.4)	1129 (38.4)	0.999
No	87 (61.3)	1725 (61.6)	1812 (61.6)	
General health score, n (%)				
Excellent	18 (12.7)	343 (12.3)	361 (12.3)	
Very good	44 (31.0)	1062 (37.9)	1106 (37.6)	0.250
Good	57 (40.1)	1008 (36.0)	1065 (36.2)	
Fair	17 (12.0)	327 (11.7)	344 (11.7)	
Poor	6 (4.2)	59 (2.1)	65 (2.2)	
Family cancer history, n (%)				
Yes	126 (88.7)	2099 (75.0)	2225 (75.7)	<0.01
No	9 (6.3)	504 (18.0)	513 (17.4)	
Not sure	7 (4.9)	196 (7.0)	203 (6.9)	
Ever had cancer, n (%)				
Yes	50 (35.2)	423 (15.1)	473 (16.1)	<0.01
No	92 (64.8)	2376 (84.9)	2468 (83.9)	

**Table 2:** Information sources about genetic testing in all study participants and participants stratified by uptake of genetic testing.

Characteristics	Did Genetic Testing (n = 142)	Did Not (n = 2799)	Total (n = 2941)	*p* Value
Information source				
Newspaper, n (%)				
Yes	29 (20.4)	417 (14.9)	446 (15.2)	0.095
No	113 (79.6)	2382 (85.1)	2495 (84.8)	
Magazine, n (%)				
Yes	31 (21.8)	493 (17.6)	524 (17.8)	0.242
No	111 (78.2)	2306 (82.4)	2417 (82.2)	
Radio, n (%)				
Yes	17 (12.0)	464 (16.6)	481 (16.4)	0.183
No	125 (88.0)	2335 (83.4)	2460 (83.6)	
Healthcare provider, n (%)				
Yes	85 (59.9)	485 (17.3)	570 (19.4)	<0.01
No	57 (40.1)	2314 (82.7)	2371 (80.6)	
Genetic counsellor, n (%)				
Yes	39 (27.5)	66 (2.4)	105 (3.6)	<0.01
No	103 (72.5)	2733 (97.6)	2836 (96.4)	
Family member, n (%)				
Yes	55 (38.7)	1032 (36.9)	1087 (37.0)	0.712
No	87 (61.3)	1767 (63.1)	1854 (63.0)	
Friend, n (%)				
Yes	38 (26.8)	797 (28.5)	835 (28.4)	0.729
No	104 (73.2)	2002 (71.5)	2106 (71.6)	
Social media, n (%)				
Yes	42 (29.6)	921 (32.9)	963 (32.7)	0.464
No	100 (70.4)	1878 (67.1)	1978 (67.3)	
Television, n (%)				
Yes	73 (51.4)	1769 (63.2)	1842 (62.6)	<0.01
No	69 (48.6)	1030 (36.8)	1099 (37.4)	
Internet, n (%)				
Yes	74 (52.1)	1369 (48.9)	1443 (49.1)	0.510
No	68 (47.9)	1430 (51.1)	1498 (50.9)	

**Table 3:** Perceptions of genetic testing in all study participants and participants stratified by uptake of genetic testing.

Characteristics	Did Genetic Testing (n = 142)	Did Not (n = 2799)	Total (n = 2941)	*p* Value
Perception				
Preventing cancer, n (%)				<0.001
A lot	91 (64.1)	1285 (45.9)	1376 (46.8)	
Somewhat	38 (26.8)	949 (33.9)	987 (33.6)	
A little	9 (6.3)	395 (14.1)	404 (13.7)	
Not at all	4 (2.8)	170 (6.1)	174 (5.9)	
Detecting cancer, n (%)				<0.001
A lot	105 (73.9)	1515 (54.1)	1620 (55.1)	
Somewhat	32 (22.5)	868 (31.0)	900 (30.6)	
A little	3 (2.1)	297 (10.6)	300 (10.2)	
Not at all	2 (1.4)	119 (4.3)	121 (4.1)	
Treating cancer, n (%)				<0.001
A lot	88 (62.0)	1334 (47.7)	1422 (48.4)	
Somewhat	32 (22.5)	884 (31.6)	916 (31.1)	
A little	9 (6.3)	394 (14.1)	403 (13.7)	
Not at all	13 (9.2)	187 (6.7)	200 (6.8)	

## Data Availability

Data and survey instruments are available on https://hints.cancer.gov/, accessed on 18 November 2022.
